# Resistance to antimicrobial drugs in different surface waters and wastewaters of Guadeloupe

**DOI:** 10.1371/journal.pone.0173155

**Published:** 2017-03-02

**Authors:** Stéphanie Guyomard-Rabenirina, Celia Dartron, Mélanie Falord, Syndia Sadikalay, Célia Ducat, Vincent Richard, Sébastien Breurec, Olivier Gros, Antoine Talarmin

**Affiliations:** 1 Unité Environnement Santé, Institut Pasteur de la Guadeloupe, Les Abymes, Guadeloupe; 2 Sorbonne Universités, UPMC Univ Paris 06, Univ Antilles, Univ Nice Sophia Antipolis, CNRS, Evolution Paris Seine—Institut de Biologie Paris Seine (EPS—IBPS), Paris, France; 3 Institut Pasteur de Nouvelle Calédonie BP61—98845 Nouméa Cedex, Nouvelle-Calédonie; 4 Université des Antilles, Pointe à Pitre cedex, Guadeloupe; 5 Laboratoire de Microbiologie Clinique et Environnementale, Centre Hospitalier Universitaire de Pointe à Pitre, Guadeloupe; Nankai University, CHINA

## Abstract

**Objective:**

The first aim of this study was to evaluate the antimicrobial resistance of *Enterobacteriaceae* in different water environments of Guadeloupe and especially those impacted by waste water treatment plants (WWTP) effluents.

The second objective was to characterize the genetic basis for antibiotic resistance of extended-spectrum beta-lactamase (ESBL) and AmpC beta-lactamase producing *Enterobacteriaceae* isolates (ESBLE and AmpCE).

**Methods:**

We have collected 70 surface waters (river and sea samples) impacted or not by WWTP and 18 waste waters from 2 WWTPs in 2012 and 2013. We i) determined the total and resistant bacterial counts and ii) tested isolated *Enterobacteriaceae* for their antimicrobial susceptibility. We also studied the genetic basis for antibiotic resistance of ESBLE and AmpCE, and the genetic background of *Escherichia coli*.

**Results:**

In rivers, contamination with *Escherichia coli* and antibiotic resistant coliforms (ARC) increased from the source to the mouth. Highest levels of river contamination with *E*. *coli* (9.26 x 10^5^ MPN/100mL) and ARC (2.26 x 10^8^ CFU/mL) were observed in surface water sampled near the discharge. A total of 246 *Enterobacteriaceae* strains resistant to antibiotics were isolated, mostly from waste waters and from river water collected near the discharge. Among these strains, 33 were Extended Spectrum Beta Lactamase (ESBLE) and 8 *E*. *coli* were AmpC beta-lactamase producers. All the ESBLE were isolated from waste waters or from river water collected near the discharge. The *bla*_CTX-M_ gene was present in 29 of the 33 ESBLE strains, with 24 belonging to group 1. Numerous strains (68.7%) showed more than one acquired antibiotic resistance mechanism. *E*. *coli* strains belonged to different phylogenetic groups; among the B2 group, most strains belonged to the ST131 clone.

**Conclusion:**

Our results demonstrated that many human activities can supply antibiotic-resistant bacteria in surface water. Nevertheless, WWTPs were the most important supplier of ESBLE in water environment of Guadeloupe.

## Introduction

Bacterial multidrug resistance remains a major public health problem worldwide, both in developed and developing countries. It results in approximately 700,000 deaths due to antibiotic-resistant bacterial infections each year [1]. In high-income countries, the continued high rates of antibiotic use in hospitals, the community, and agriculture have been suspected as one of the main reasons for the spread of multiresistant bacteria [2]. *Enterobacteriaceae*, particularly *Escherichia coli*, are subject to this selective pressure, as the digestive tract of human and warm-blooded animals is their main reservoir. One major problem is the resistance mediated by acquired extended-spectrum beta-lactamase (ESBL) genes carried by the transfer of genes present in mobile genetic elements (integrons, transposons, plasmids) [3]. This is of great concern, as ESBL enzymes can hydrolyse nearly all beta-lactams (except carbapenems and cephamycins). In addition, they are frequently associated with genes that confer resistance to several other classes of antibiotics, complicating the first-line treatment for many infections. CTX-M enzymes have become the most prevalent ESBLs in many countries, both in nosocomial and in community settings, replacing the classical TEM and SHV-type ESBLs in many countries [4]. Plasmids carrying *bla*_CTX-M_ genes frequently carry other antibiotic resistance determinants such as plasmid-mediated quinolone resistance [5]. In *E*. *coli*, they are frequently carried in well-adapted phylogenetic groups with particular virulence-factor genotypes [6]. The spread of successful resistant clones and plasmids may be responsible for the increase in antibiotic resistance worldwide. This is highlighted by the global dissemination of sequence type 131 (ST131) *E*. *coli* harbouring *bla*_CTX-M-15_ on incompatibility group FII conjugative plasmids [7].

In the community, antibiotic resistant bacteria (ARB) spread easily through the environment or between humans, particularly in low-income countries [8,9]. Poor hygiene is suspected to be the main reason for this transmission. However, bacterial strains of fecal origin such as resistant *E*. *coli* or *E*. *coli* O25b-ST131 can also spread in high-income countries, despite a good level of hygiene [10,11]. Thus, other sources of transmission of ARB must be identified. Environmental dissemination is likely, especially through wastewater [12]. Wastewater is rich in nutrients, antimicrobial substances, and other pollutants such as heavy metals, and thus offer optimal conditions for bacterial development and the spread of ARB by mutation or horizontal gene transfer [13,14]. Although wastewater treatment plant significantly reduces the total number of bacteria [15], numerous studies have demonstrated that treated wastewater may contain ARB, especially ESBL producing *Enterobacteriaceae* and contribute to the contamination of surface water with ARB [16–20].

Guadeloupe, a French overseas territory located in the Caribbean, is a very high-resource country according to the Human Development Index in 2013 (http://hdr.undp.org/).)Guadeloupe was called “The Island of Beautiful Waters” by the indigenous Arawak people due to the numerous rivers and waterfalls lining the “Basse-Terre”, one of the two main islands of Guadeloupe. Unfortunately, the low performance of the wastewater collection and treatment systems, as well as the industrial and agricultural effluents, are responsible for the pollution of surface water and groundwater [21].

Before this study, very little was known concerning resistance to antibiotics in humans and no data existed concerning resistance in the environment in Guadeloupe. Since rivers are present all over the “Basse-Terre” and the housing rather dense, the probability of contamination of surface waters by human activities is rather high. To better appreciate the contamination of surface waters by antibiotic resistant coliforms, we undertook the following study.

The first objective of this study was to evaluate the antimicrobial resistance of *Enterobacteriaceae* in surface waters such as rivers, sea and WWTPs effluents, in Guadeloupe. The second objective was to characterize the genetic basis for antibiotic resistance of ESBL and AmpC producing *Enterobacteriaceae* isolates, as well as the genetic background of *E*. *coli* to determine whether the isolate belonged to the successful and hypervirulent *E*. *coli* O25b-ST131 group and to evaluate whether strains harbouring ESBLs belonged to phylogenetic groups more specific to human infections.

## Material and methods

### Sample collection

We collected 70 surface waters and 18 wastewaters from WWTPs between 2012 and 2013. Sampling was performed during the dry (February to May) and the rainy (October to December) seasons.

For the waste water treatment plants authorizations were obtained from the WWTPs directors.

For surface waters, no specific permissions were required for these sampling locations since they are in public lands.

**Fig 1:**
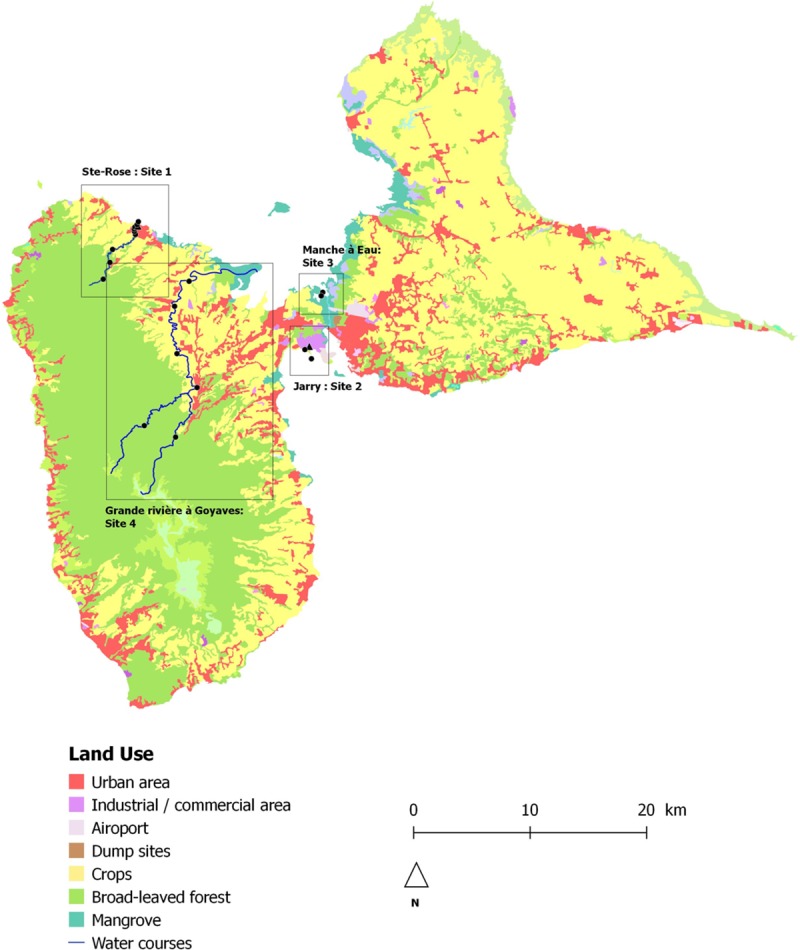
Map of Guadeloupe and location of the sampling sites.

**Fig 2:**
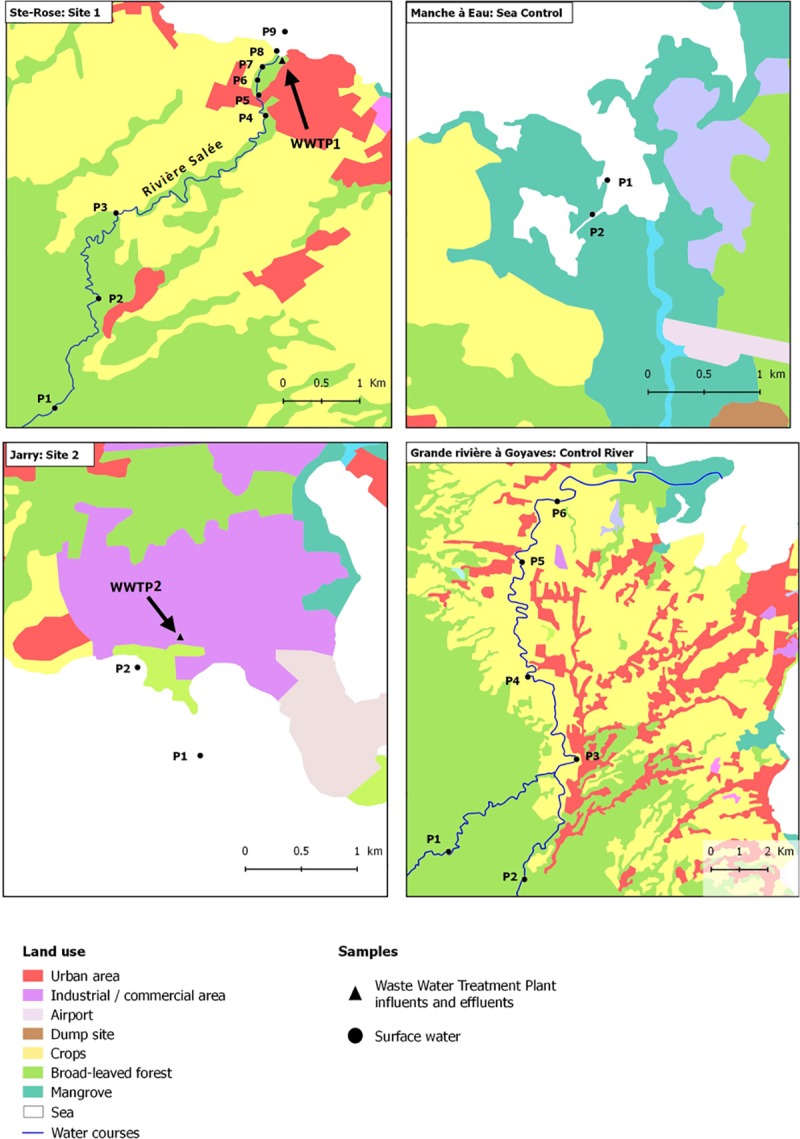
Sampling locations in different sites.

River samples were collected along the course of 2 rivers of Guadeloupe. Sea samples were collected at the mouth of the rivers, in the open sea and in the mangrove (Figs 1 and 2). Each location was sampled two to six times during the course of the study (Table 1).

**Table 1:** Number of *E*. *coli*, total and antibiotic resistant coliforms.

					No. of *E*. *coli* strains (MPN/100ml)				Bacterial count on L-TTC +/- antibiotics (CFU/mL)					
Site	Point of sampling	Geographic coordinates	Land use around sampling point	Nb of sampling	Mean Number	Min—max	L-TTC		L-TTC + AMP		L-TTC + CAZ		L-TTC + CIP	
							Mean number	Min—max	Mean number (%)	Min—max	Mean number (%)	Min—max	Mean number	Min—max
**1**	WWTP Influent	16°20'02"N 61°42'04"W		6	2.38 x 10^7^	6.01 x 10^5^–8.20 x 10^7^	6.80 x 10^8^	2.52 x 10^7^–2.38 x 10^9^	3.4 x 10^8^ (50.00)	1.50 x 10^7^–1.20 x 10^9^	1.22 x 10^8^ (17.9)	3.65 x 10^6^–4.32 x 10^8^	1.10 x 10^7^ (1.6)	3.98 x 10^5^–3.90 x 10^7^
	WWTP Effluent			6	7.62 x 10^5^	4.01 x 10^4^–2.17 x 10^6^	9.00 x 10^7^	4.15 x 10^6^–3.26 x 10^8^	4.8 x 10^7^ (53.3)	4.25 x 10^6^–1.68 x 10^8^	2.10 x 10^7^ (23.3)	8.30 x 10^5^ -7.07 x 10^7^	1.00 x 10^6^ (1.1)	3.80 x 10^4^–3.70 x 10^6^
	Point 1	16°17'29''N 61°43'44"W	Broad-leaved forest	2	0	0.00	3.61 x 10^4^	1.27 x 10^4^–5.95 x 10^4^	9.20 x 10^3^ (25.5)	5.60 x 10^3^–1.28 x 10^4^	3.45 x 10^2^ (1.1)	1.10 x 10^2^–3.90 x 10^2^	40.00 (0.1)	20.00–60.00
	Point 2	16°18'16"N 61°43'23"W		2	7.50	0.00–15	1.07 x 10^4^	6.50 x 10^3^–1.4 9x 10^4^	5.00 x 10^3^ (46.7)	2.42 x 10^3^–7.58 x 10^3^	1.00 x 10^3^ (9.3)	0–2.00 x 10^3^	0.00 (0.0)	0.00
	Point 3	16°18'55"N 61°43'17"W		2	63.50	0.00 -127	2.60 x 10^4^	2.00 x 10^4^–3.20 x 10^4^	1.84 x 10^4^ (70.8)	1.22 x 10^4^–1.84 x 10^4^	2.10 x 10^3^ (8.2)	1.10 x 10^3^–3.10 x 10^3^	0.00 (0.0)	0.00
	Point 4	16°19'38"N 61°42'11"W	Urban area/Crops	2	2.42 x 10^2^	1.60 x 10^2^–3.24 x 10^2^	4.40 x 10^4^	8.00 x 10^3^–8.00 x 10^4^	3.08 x 10^4^ (70.00)	2.70 x 10^4^–3.84 x 10^4^	2.30 x 10^3^ (5.4)	5.00 x 10^2^–4.10 x 10^3^	40.00 (0.09)	0–80
	Point 5	16°19'47"N 61°42'14"W		2	9.08 x 10^3^	8.32 x 10^3^–9.83 x 10^3^	1.40 x 10^5^	8.00 x 10^4^ -2.00 x 10^5^	3.92 x 10^4^ (35.7)	3.84 x 10^4^–4.00 x 10^4^	3.10 x 10^4^ (22.2)	2.90 x 10^4^–3.30 x 10^4^	2.20 x 10^3^ (1.6)	1.50 x 10^3^–3.00 x 10^3^
	Point 6	16°19'54''N 61°42'14''W		6	2.30 x 10^5^	2.45 x 10^4^–3.99 x 10^5^	8.50 x 10^5^	2.25 x 10^4^–2.30 x 10^6^	7.10 x 10^5^ (83.7)	4.00 x 10^5^–1.60 x 10^6^	3.98 x 10^5^ (46.00)	5.90 x 10^4^–1.31 x 10^6^	1.38 x 10^5^ (16.1)	1.6 x 10^4^–2.00 x 10^5^
	Point 7	16°20'07''N 61°42'15''W	Discharge	6	9.26 x 10^5^	8.55 x 10^4^–4.01 x 10^6^	2.70 x 10^6^	3.10 x 10^5^–1.70 x 10^7^	2.28 x 10^6^ (84.4)	3.60 x 10^5^–4.00 x 10^6^	6.40 x 10^5^ (23.6)	1.60 x 10^5^–1.37 x 10^6^	3.82 x 10^5^(14.0)	2.00 x 10^4^–8.00 x 10^5^
	Point 8	16°20'06"N 61°42'03"W	Mangrove	6	8.68 x 10^3^	1.35 x 10^3^–4.09 x 10^4^	2.85 x 10^5^	5.70 x 10^4^–1.70 x 10^6^	2.70 x 10^5^ (94.7)	1.00 x 10^3^–3.80 x 10^5^	2.25 x 10^5^ (78.9)	1.60 x 104–6.20 x 105	2.40 x 10^4^ (8.4)	9.30 x 10^3^–5.30 x 10^4^
	Point 9	16°20'15"N 61°42'02"W	Open sea	6	1.88 x 10^3^	0–6.39 x 10^3^	5.75 x 10^4^	1.30 x 10^4^–2.70 x 10^5^	2.60 x 10^4^ (45.2)	4.00 x 10^2^–4.00 x 10^4^	2.60 x 10^3^ (4.5)	1.00 x 10^2^–5.50 x 10^3^	4.50 x 10^2^ (0.8)	0.00–1.3 x 10^3^
**2**	WWTP Influent	16°14'15"N 61°33'49"W		3	1.21 x 10^7^	8.93 x 10^6^–1.76 x 10^7^	4.00 x 10^8^	2.03 x 10^8^–7.41 x 10^8^	2.30 x 10^8^ (57.5)	1.52 x 10^8 _^ 3.85 x 10^8^	6.2 x 10^7^ (15.5)	3.80 x 10^7^–1.02 x 10^8^	7.70 x 10^6^ (1.9)	3.75 x 10^6^–1.50 x 10^7^
	WWTP Effluent			3	3.93 x 10^5^	1.34 x 10^5^–8.42 x 10^5^	5.80 x 10^7^	3.58 x 10^7^–8.54 x 10^7^	2.30 x 10^7^ (39.6)	1.52 x 10^7^–3.49 x 10^7^	5.2 x 10^5^ (9.00)	4.32 x 10^5 _^ 6.16 x 10^5^	4.00 x 10^5^ (0.7)	2.35 x 10^5^–5.28 x 10^5^
	Point 1	16°13'39"N 61°33'43" W	Discharge	6	14.1	<15–30	7.00 x 10^2^	3.00 x 10^2^–9.00 x 10^2^	5.00 x 10^2^ (71.4)	20.00–2.00 x 10^3^	62.00 (8.8)	20.00–1.00 x 10^2^	1.00 x 10^2^ (14.3)	0.00–6.70 x 10^2^
	Point 2	16°14'07"N 61°34'00"W	Broad-leaved forest	6	10	<15	7.30 x10^3^	3.00 x 10^2^–4.12 x 10^4^	3.01 x 10^3^ (42.4)	60.00–1.00 x 10^4^	2.1 x 10^3^ (28.8)	2.5 x 10^2^–1.3 x 10^4^	2.20 x 10^2^ (3.00)	0.00–5.20 x 10^2^
**3**	Point 1	16°16'51"N 61°33'10"W	Mangrove	6	67	<15–3.47 x 10^2^	1.10 x 10^3^	3.00 x 10^2^ -1.45 x 10^4^	8.00 x 10^2^ (72.7)	4.20 x 10^2^–2.60 x 10^3^	1.3 x 10^2^ (11.8)	50.00–1.00 x 10^3^	80.00 (7.3)	40.00–2.50 x 10^2^
	Point 2	16°16'41"N 61°33'15"W		6	24.33	< 15–61	6.30 x 10^3^	3.50 x 10^3^–2.60 x 10^4^	1.60 x 10^3^ (25.3)	3.00 x 10^2^–5.00 x 10^3^	8.1 x 10^2^ (12.8)	1.70 x 10^2^–2.00 x 10^3^	1.70 x 10^2^ (2.7)	40.00–6.00 x 10^2^
**4**	Point 1	16°10'27''N 61°41'45"W	Broad-leaved forest	2	0	0	8.00 x 10^3^	6.00 x 10^3^–10.00 x 10^3^	4.98 x 10^3^ (62.25)	1.00 x 10^3^–8.96 x 10^3^	6.2 x 10^2^ (7.75)	1.00 x 10^2^–9.40 x 10^2^	0.00 (0.0)	0.00
	Point 2	16°09'53''N 61°40'15"W		2	2.40 x 10^2^	0–4.80 x 10^2^	2.35 x 10^4^	9.00 x 10^3^–4.69 x 10^4^	1.78 x 10^4^ (76.2)	5.60 x 10^3^–3.00 x 10^4^	3.5 x 10^3^ (14.9)	1.70 x 10^3^–5.30 x 10^3^	0.00 (0.0)	0.00
	Point 3	16°12'16"N 61°39'13"W	Urban area/Crops	2	4.52 x 10^3^	0–9.04 x 10^3^	6.05 x 10^4^	2.10 x 10^4^–1.00 x 10^5^	4.05 x 10^4^ (66.9)	4.00 x 10^4^–4.50 x 10^4^	1.37 x 10^4^ (22.6)	1.14 x 10^4^–1.6 x 10^4^	50.00 (0.16)	0.00–1.00 x 10^2^
	Point 4	16°13'54"N 61°40'11"W		2	93.5	30–1.27 x 10^2^	6.00 x 10^4^	2.00 x 10^4^–1.00 x 10^5^	4.15 x 10^4^ (69.2)	3.50 x 10^3^–4.80 x 10^4^	1.68 x 10^4^ (28.0)	3.00 x 10^3^–3.33 x 10^4^	0.00 (0.0)	0.00
	Point 5	16°16'09"N 61°40'18"W		2	1.81 x 10^2^	46–2.70	7.00 x 10^4^	3.00 x 10^4^–1.10 x 10^5^	5.00 x 10^4^ (71.4)	2.00 x 10^4^–8.00 x 10^4^	4.45 x 10^3^ (15.7)	3.40 x 10^3^–5.50 x 10^3^	1.00 x 10^2^ (0.14)	26.00–1.74 x 10^2^
	Point 6	16°17'23"N 61°39'36"W		2	2.10 x 10^2^	93–2.34 x 10^2^	7.00 x 10^4^	6.00 x 10^4^–8.00 x 10^4^	5.00 x 10^4^ (71.4)	4.00 x 10^4^–5.00 x 10^4^	5.00 x 10^3^ (7.1)	3.90 x 10^3^–6.10 x 10^3^	3.36 x 10^2^ (4.8)	1.70 x 10^2^–5.02 x 10^2^

WWTP, waste water treatment plants; MPN, most probable number; CFU, colony-forming unit; Selective growth media: TTC Triphenyltetrazolium Chloride; AMP, ampicillin; CAZ, ceftazidime; CIP, ciprofloxacin

River at Site #1 was impacted by effluents from a WWTP and one WWTP discharged directly into the sea (Site #2). The mangrove in #Site 3 and the river in #Site 4 were not connected to the WWTPs effluents (Figs 1 and 2).

At Site #1 (Ste Rose), samples from influents and effluents were collected from the WWTP1; samples from the “Rivière Salée” were collected at 7 points: one point at the source (P1), four points along the river (P2–P5), one point 500 m upstream of the discharge (P6), and one point at the discharge (P7). Seawater samples were also collected near the mouth of the river (P8) and from the open sea (P9). The source of this river is located on the North side of Basse-Terre at an altitude of 400 m, in the woods. This river then flows to the sea between houses that can be seen from point 2, crosses fields used to cultivate sugar cane or raise cattle and end in the small town of Sainte-Rose (Fig 2).

At Site #2 (Jarry), apart from influent and effluent samples from WWTP2, seawater samples were taken at two sampling points: one near the discharge (P1) and one near the mangrove (marine environment) (P2) (Fig 2).

At Site #3, water samples were collected at two points in “Manche à Eau” (in the lagoon (P1) and near the mangrove (P2)).

At Site #4, river water samples from “La Grande Rivière à Goyave” were taken at six points from the source to the mouth (P1–P6). This river rises from the east side of Basse-Terre at an altitude of 500 m and crosses through its itinerary to the sea mainly agricultural fields (sugar cane and cattle) and some small villages from point 3 (Fig 2).

### Bacterial counts, isolation, and antibiotic susceptibility test

The most probable number (MPN) of *E*. *coli* was determined using MUG microplates (Bio-Rad, Marnes-la-Coquette, France). We used the membrane filtration (MF) lactose Tergitol-7 method (LTTC) (ISO 9308-1:200) to count the number of total and antibiotic resistant coliforms (ARC). First, 100 ml of water samples serially diluted tenfold in physiological saline were filtered through 0.45 μm membrane filters (Millipore, Guyancourt, France). The filters were placed on presumptive LTTC for *Enterobacteriaceae* with antibiotics (2 mg/L ampicillin; 2 mg/L cefotaxime; 1 mg/L ciprofloxacin) [22] and without antibiotics in order to determine the percentage of resistant *Enterobacteriaceae*. The plates were incubated for 24-48 h at 37°C. The percentage of ARC was calculated by dividing the number of total bacteria growing on LTTC with antibiotics by the number of bacteria growing on LTTC media without antibiotics. Presumptive *Enterobacteriaceae* colonies on LTTC (orange colonies, oxidase negative, Gram-negative bacilli) were randomly isolated and identified using the Api 20 E test (BioMérieux, Marcy-l-Étoile, France). Three colonies were randomly identified for each identical morphology; all colonies that differed by morphology were selected. The susceptibility of the *Enterobacteriaceae* strains to antibiotics was assessed using the disk diffusion technique on Mueller-Hinton agar as recommended by the Antibiogram Committee of the French Microbiology Society [22]. Strains were tested against different antimicrobial agents: amoxicillin (10 μg), amoxicillin-clavulanic acid (20 μg/10 μg), ticarcillin (75 μg), cephalothin (30 μg), cefotaxime (30 μg), ceftazidime (30 μg), cefoxitin (30 μg), aztreonam (30 μg), imipenem (10 μg), gentamicin (15 μg), amikacin (30 μg), trimethoprim/sulfamethoxazole (1.25/23.75 μg), nalidixic acid (30 μg), ciprofloxacin (5 μg), and tetracycline (30 UI). Extended-spectrum betalactamases (ESBL) were detected by the combined disk method using cefotaxime (30 μg) and ceftazidime (30 μg) *versus* cefotaxime plus clavulanate (30 and 10 μg) and ceftazidime plus clavulanate (30 and 10 μg). Growth inhibition diameters were measured using the Adagio automated system (Bio-Rad). Intermediate and resistant *Enterobacteriaceae* strains were grouped together and classified as resistant strains. *Escherichia coli* ATCC 25922 was used as a control strain.

The minimal inhibitory concentration (MIC) of imipenem was determined using the E-test (Biomérieux) for strains exhibiting reduced susceptibility to this antibiotic.

Only one randomly chosen isolate was analysed if more than one strain belonging to the same species, with the same antibiotic susceptibility pattern, was isolated from the same sampling point at the same time.

### Molecular characterization of antibiotic resistance genes

Total DNA was extracted using the Nucleospin tissue kit according to the manufacturer’s instructions. (Macherey Nagel, Hoerdt, France). Previously described PCR methods were used to screen for plasmid-encoded *bla*_*CTX-M*_, *bla*_TEM_, and *bla*_SHV_ betalactamase [23–25]. Isolates resistant to cefoxitin were tested for the presence of six families of plasmid-borne *ampC* genes (FOX, ACC, EBC, MOX, CIT, and DHA) [26]. Strains resistant to imipenem (MIC > 8 mg/L) were tested for the presence of carbapenemase-encoding genes (*bla*_VIM_, *bla*_KPC_, *bla*_IMP_, *bla*_NDM_, and *bla*_*OXA-48*_) [27]. Beta-lactamases were then characterised by direct DNA sequencing of the PCR products (Eurofins Genomic SAS, Les Ullis, France). The nucleotide sequences and deduced protein sequences were analysed using the BLAST, ResFinder, and Clustal W programs. All ESBLE resistant to ciprofloxacin were screened by PCR amplification for the presence of the *qnrA*, *qnrB*, *qnrS* genes [28].

### Phylogenetic group determination of *E*. *coli* strains

Phylogrouping of ESBL producing *E*. *coli* strains was based on triplex PCR (*chuA*, *yjaA*, and TspE4.C2) [29]. Duplex PCR targeting of the *pabB* and *trpA* genes was used to determine whether the isolate belonged to the O25b-ST131 group [30].

### Data analysis

R software was used for statistical analysis [31]. We used Kruskal-Wallis and chi-square tests to compare numeric data and percentages, respectively. We used the Spearman nonparametric test to analyse the correlation between the distance of the sample site to the mouth of the river and number of bacteria in the sample. The distance from the mouth of the river was used as a marker of anthropization, as in Guadeloupe, anthropization globally increases from the inland to the coast (Fig 1).

## Results

### Abundance of total and resistant bacteria in the studied samples

In surface waters, *E*. *coli* concentrations ranged from 0 in samples collected at the sources of the rivers to 9.26 x 10^5^ MPN/100 mL in samples collected near the WWTP discharge at Site #1. The mean *E*. *coli* MPN found in the different water samples collected near the discharges of the WWTPs were significantly lower in sea water at Site #2 (14 per 100 mL) than in river water at Site # 1 (9.26 x10^5^ per 100 mL) (p < 0.01). For ARC, the total numbers of bacteria resistant to the various antibiotics tested were one hundred times lower in the samples collected from the discharge at Site #2 in the sea than in those from the discharge in river #1; however, the percentage of ARC was not significantly different (Table 1).

In river at Site #1, there was a significant inverse correlation between the level of contamination with *E*. *coli* and ARC, and the distance between the sample point and mouth of the river 1 (p < 0.01). In the river at Site #4, although the number of bacteria on LTTC increased from the source to the mouth, the difference was not significant. Globally, bacterial contamination increased with the anthropization of the landscape (Fig 1, Table 1). Near the sources that are located in the natural landscape, mainly tropical forest, contamination with *E*. *coli* and ARC was very low. The rivers then cross small villages and agricultural fields with cattle, where contamination with *E*. *coli* and ARC increased. For the river at Site #4, housing remain scarce, whereas Ste Rose at Site # 1 is a town with 20,000 inhabitants. Only a few industrial areas exist along the rivers.

In contrast to the percentage of bacteria resistant to ampicillin, which was rather stable at all sampling points, the percentage of bacteria resistant to ceftazidime or ciprofloxacin was significantly higher in areas under anthropic influence. Indeed, the percentage increased along the “Rivière Salée” and the “Grande Rivière à Goyave” with the level of anthropization in the landscape (Figs 1 and 2, Table 1).

Seawater samples collected downstream of the discharge at Site #1 showed a substantial reduction in bacterial contamination rates (Table 1).

The median *E*. *coli* MPN counts were significantly lower in the effluent than in the influent for both WWTPs (p < 0.01), with a mean reduction of 96.8%. (Table 1). The percentage of antimicrobial-resistant bacteria was significantly different between the influents and effluents of both plants (p < 0.01). Lower bacterial resistance was observed against ampicillin, ceftazidime, and ciprofloxacin for the WWTP 2 effluents. Conversely, for WWTP 1 effluents, higher resistance was observed against ampicillin and ceftazidime; however, lower resistance was observed against ciprofloxacin (Table 1).

### Characterization of antimicrobial resistant isolates

**Fig 3:**
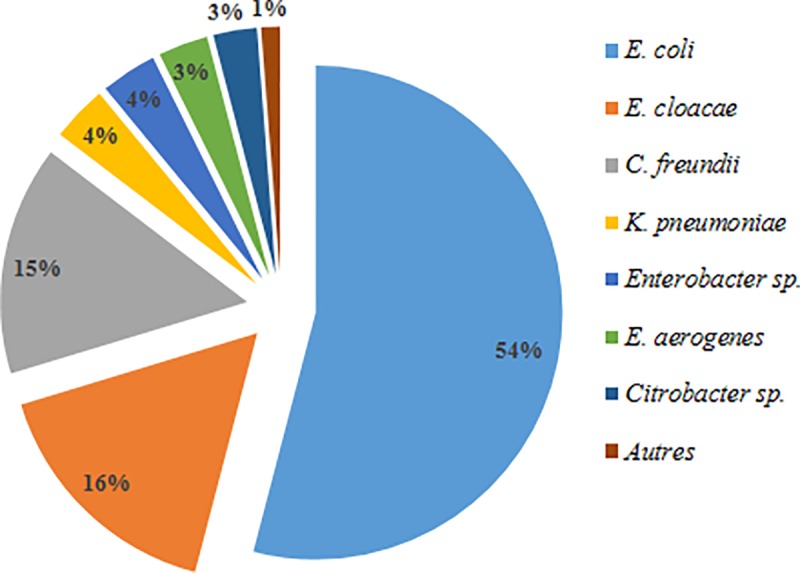
Distribution of species among *Enterobacteriaceae* strains.

**Fig 4:**
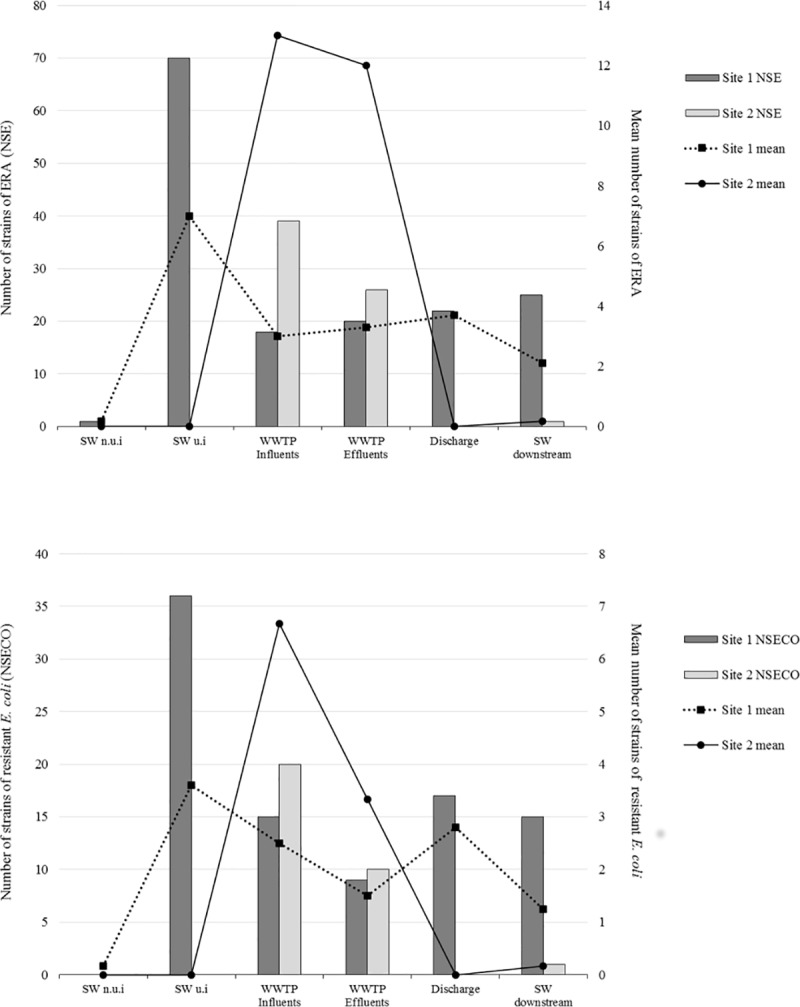
**Total and mean number of strains of *Enterobacteriaceae* resistant to antibiotics (A) and total and mean number of strains of *E*. *coli* resistant to antibiotics (B) in surface water and waste water**.in Site #1 and Site #2 SW = Surface water, n.u.i = non under anthropic influence (sampling point surrounded by broad leaved forest); u.i = under anthropic influence (sampling point surrounded by urban areas or crops).

We analysed 246 randomly selected *Enterobacteriaceae* strains resistant to antibiotics (ARE), of which 54% were *Escherichia coli*. Other frequently encountered species were *Enterobacter cloacae* (16.3%) and *Citrobacter freundii* (15%) (Fig 3). We isolated a high percentage (41.9%) of ARE strains from WWTPs influents and effluents. We isolated similar numbers of resistant strains from raw and treated water at WWTP1, whereas we isolated 40 ARE strains from raw water and 26 from treated water at WWTP2. The number of multiresistant bacteria was proportionally higher in the effluent than in the influent of both WWTPs (Fig 4A and 4B).

The 143 remaining ARE strains were mostly isolated at Site #1 (118, 82.5%). Most (73.7%) of them were isolated from samples collected near and 500 m upstream of the discharge (23.2% and 50.5%, respectively). At this site, the mean number of ARE was higher in the surface water than in WWTPs influents and effluents (Fig 4A and 4B).

At Site#3 and Site #4, only 24 ARE strains could be isolated, 8 in areas non under anthropic influence and 16 in areas under anthropic influence.

**Fig 5:**
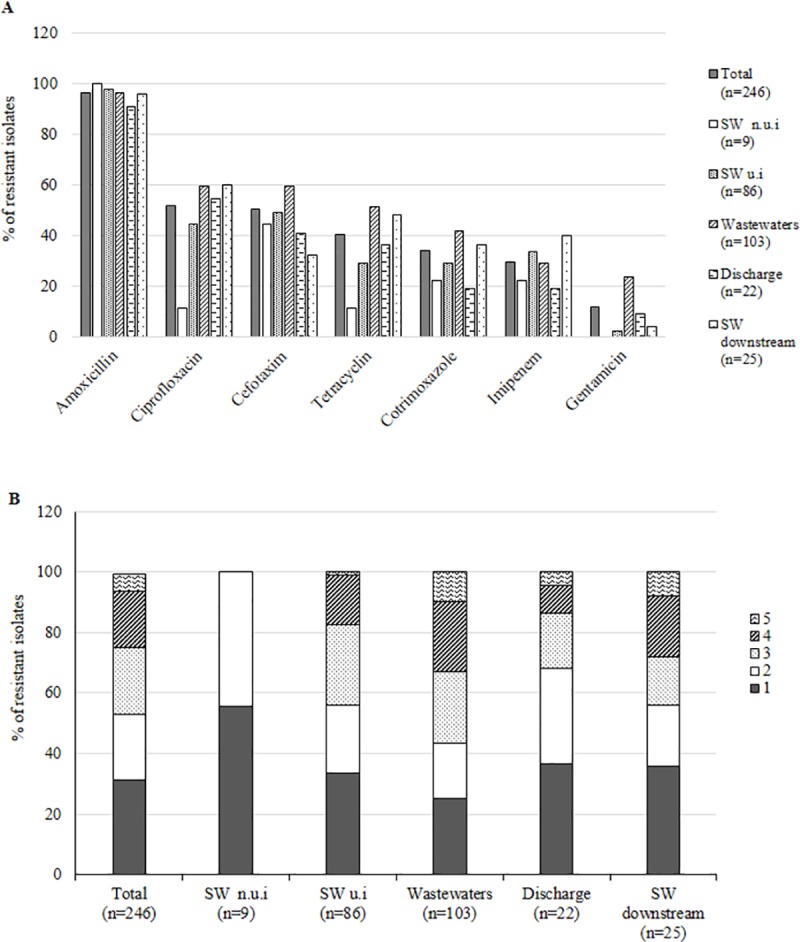
Resistance phenotypes of *Enterobacteriaceae* strains isolated from surface waters and wastewaters. Shown are the percentages of isolates resistant to each class of antibiotic tested (A) and percentages of isolates resistant to different numbers of antibiotic classes (B). n.u.i = non under anthropic influence (sampling point surrounded by broad leaved forest); u.i = under anthropic influence (sampling point surrounded by urban areas or crops).

Among the 246 ARE isolates, 124 (50.4%) were resistant to third-generation cephalosporins (3GC) (47 *Enterobacter* sp., 30 *E*. *coli*, 39 *Citrobacter* sp., 7 *Klebsiella pneumoniae*, and 1 *Serratia liquefasciens*). In addition, *Enterobacteriaceae* isolates showed high rates of resistance to ciprofloxacin (51.6%), cotrimoxazole (34.1%), and tetracycline (40.2%), whereas resistance to gentamicin was observed in only 11.8% of the isolates (Fig 5A). Higher rates of resistance to ciprofloxacin, tetracyclin and gentamicin were observed among the isolates obtained from wastewaters and surface water impacted by WWTP effluents (Fig 5A). One strain of *E*. *cloacae* was resistant to imipenem (CMI > 32 μg/mL). Resistance to ciprofloxacin and cotrimoxazole was particularly high among ESBLE (51.5% and 42.4%, respectively) (Table 2).

**Table 2:** Characteristics of ESBL and AmpC β-lactamases *Enterobacteriaceae* producing strain.

Species and ESBL/AmpC β-lactamase type	*E*. *coli* phylogroup	Site of sampling		Additionnal β lactamase			Co-acquired resistance^a^				PMQR genes^b^
				non ESBL SHV	TEM1-like	OXA-1 like	SXT	GM	CIP	TE	
*E*. *coli* (27)											
CTX-M-1 (9)	A (5)	Site 1 WWTP I (2)		_	1	_	_	2	_	1	_
		Site 1 WWTP E		_	_	_	_	_	_	1	_
		Site 1 Pt 7		_	1	_	_	1	_	1	_
		Site 1 Pt 8		_	_	_	1	_	_	1	_
	B1 (2)	Site 1 Pt 7		_	1	_	1	_	_	1	_
		Site 2 WWTP I		_	_	_	_	_	_	_	_
	B2 (2)	Site 2 WWTP I		_	_	_	_	_	_	1	_
		Site 2 WWTP E		_	_	_	_	_	_	_	_
CTX-M-15 (3)	A	Site 1 Pt 7		_	_	1	1	1	1	1	_
	B2	Site 1 Pt 7		_	_	1	_	_	1	1	_
	D	Site 2 WWTP E		_	1	_	1	1	1	1	_
CTX-M-14 (2)	D	Site 2 WWTP E		_	1	_	1	_	_	1	_
	A	Site 1 Pt 6		_	_	_	_	_	_	_	_
CTX-M-27	A	Site 1 WWTP I		_	_	_	1	_	1	1	_
CTX-M-8 (2)	B1	Site 2 WWTP I (2)		_	_	_	_	_	2	_	_
SHV-12 (2)	B1	Site 1 Pt 7		_	_	_	_	_	_	_	_
	B2	Site 1 Pt 7		_	_	_	_	_	_	_	*_*
CMY-2 (7)	A (5)	Site 1 WWTP I (2)		_	1	_	_	_	_	_	*_*
		Site 1 Pt 5 (2)		_	_	_	_	_	_	_	*_*
		Site 4 Pt 6		_	_	_	_	_	_	_	*_*
	B1	Site 2 WWTP I		_	_	_	1	_	1	_	_
	D	Site 1 WWTP E		_	_	_	_	_	_	_	*_*
CMY-8	B1	Site 1 WWTP E	_	_	_	_	_	_	1	_	*_*
*K*. *pneumoniae* (5)											
CTX-M-15 (3)	_	Site 2 WWTP E (3)		2	2	1	1	2	3	3	*qnrB19 (2) qnrB1 aac(6′)-1b-cr*
VEB-1 (1)	_	Site 2 WWTP I		_	_	_	_	_	_	_	*_*
TEM-3 (1)	_	Site 2 WWTP I		_	_	_	1	_	_	1	*_*
*E*. *cloacae* (5)											
CTX-M-15 (5)	_	Site 2 WWTP I (3)		1	3	3	3	3	3	3	*qnrB19*
		Site 2 WWTP E (2)		_	1	1	1	1	2	1	_
*E*. *aerogenes* (1)											
CTX-M-15 (1)	_	Site 2 WWTP I		_	1	1	1	1	1	1	_
*E*. *amnigenus* (1)											_
CTX-M-15 (1)	_	Site 2 WWTP I		_	_	1	_	_	1	_	_
*E*. *sakazakii* (1)											
CTX-M-15 (1)	_	Site 2 WWTP I		_	1	1	1	1	1	1	_
*C*. *freundii* (1)											
CTX-M-1 (1)	_	Site 2 WWTP I		_	_	_	_	_	_	1	_
											

^a^ SXT, cotrimoxazole; GM, gentamicin; CIP, ciprofloxacin; TE, tetracyclin

^b^ PMQR, Plasmid Mediated Quinolone Resistance

Most of isolates (68.7%) were resistant to at least two classes of antibiotics. Isolates from wastewater were more resistant than isolates from surface waters (NS), ten isolates from wastewaters were resistant to the five classes of antimicrobials tested (Fig 5B). Among the nine isolates from surface water, none was resistant to more than three antimicrobials.

### Characterization of antibiotic resistance genes

We identified 33 ESBLE isolates, of which 19 were *E*. *coli*, among the 246 total ARE isolates studied. Most of ESBLE strains (25) were isolated from WWTP effluents, particularly from the WWTP 1 effluent (22 strains). All ESBLE strains obtained from surface waters were found in river at Site #1, from samples collected near or downtsream the discharges (Table 2). The *bla*_CTX-M_ gene was present in 29 of the 33 ESBLE strains, with 24 belonging to group 1 (10*bla*_CTX-M-1_ and 14 *bla*_CTX-M-15_), 3 to group 9 (2 *bla*_CTX-M-27_, 1 *bla*_CTX-M-14_), and 2 to group 8 (*bla*_CTX-M 8_)_._ Other ESBLs present were 2 SHV-12, 1 TEM-3, and 1 VEB-1; the latter was found in a *K*. *pneumoniae* strain (Table 2). We found additional β lactamases in 15 (45.4%) of the ESBLE and *qnrB* genes (3 *qnrB19* and 1 *qnrB1*) in 4 CTX-M-15-producing strains.

Eight *E*. *coli* strains resistant to 3GC and cefoxitin were AmpC β-lactamase producers; they produced only CMY enzymes, with CMY-2 produced by 7 strains. Five AmpC β-lactamase producers were isolated from wastewater and 3 from surface water collected in rivers from sampling points under anthropic influence. We identified an additional β lactamase, TEM-1, in only one strain. Co-acquired resistance occurred less frequently than in ESBL producers (Table 2).

The *E*. *cloacae* strain resistant to imipenem did not produce carbapenemase.

### Phylogenetic groups of *E*. *coli* strains

**Fig 6:**
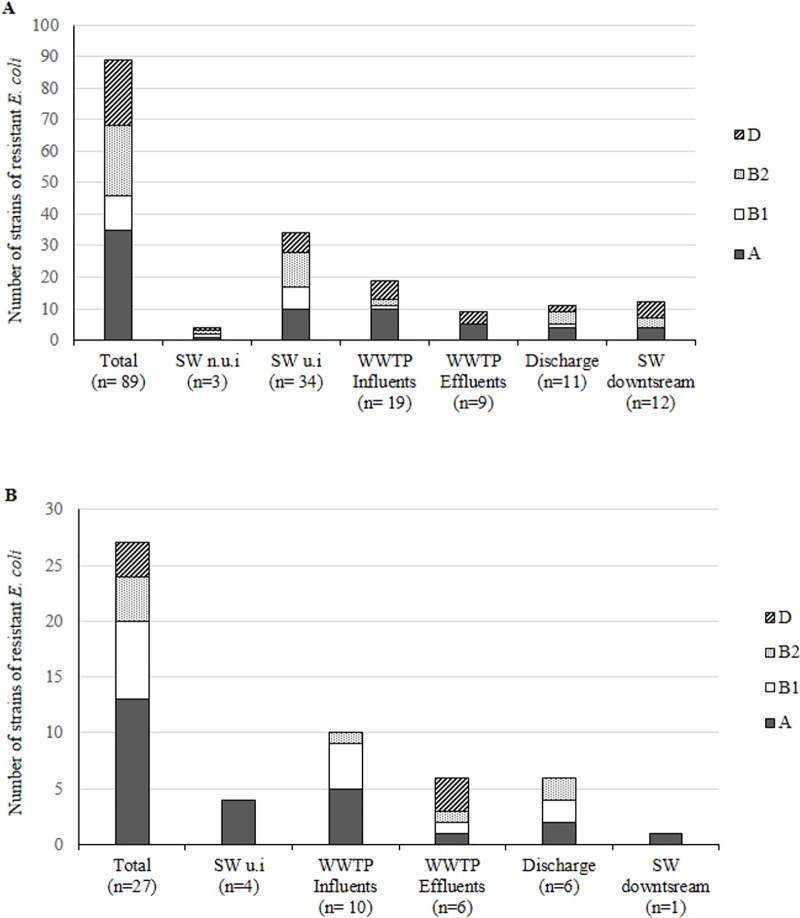
**Distribution of phylogenetic groups of *E*. *coli* in non ESBL/AmpC producing strains (A) and ESBL/AmpC producing strains (B) in surface water and waste water**. SW = Surface water, n.u.i = non under anthropic influence (sampling point surrounded by broad leaved forest); u.i = under anthropic influence (sampling point surrounded by urban areas or crops).

Phylogenetic groups were determined for 116 of the 133 *E*. *coli* strains. Most *E*. *coli* strains belonged to the commensal A and B1 groups (48 and 18 isolates, respectively). Fifty strains belonged to phylogenetic groups B2 (30) and D (24), which are known to comprise most human extra-intestinal strains. All strains belonging to the B2 group were isolated from areas under anthropic influence (Fig 6A and 6B).

The phylogenetic groups B1 and B2 were more frequent and phylogenetic group D was less frequent among *E*. *coli* ESBL-producing strains than among *E*. *coli* that did not produce ESBL; however, the differences were not significant (Fig 6A and 6B). No significant differences were observed in the distribution of the phylogenetic groups according to the type of ESBL.

Most *E*. *coli* B2 strains belonged to clone O25b-ST131 (N = 18, 69, 2%); among these, the rate of those producing ESBL was the same as that of strains that did not belong to this clone (25%).

## Discussion

The first objective of this study was to evaluate the antimicrobial resistance of *Enterobacteriaceae* in different water environments of Guadeloupe and especially those impacted by WWTPs effluents. We determined the counts of total and resistant bacteria in WWTP influents and effluents as well as those from rivers and seawater receiving or not the effluents. In rivers, percentages of ARC resistant to 3GC or fluoroquinolones were low and no or very few *Enterobacteriaceae* strains resistant to 3GC or fluoroquinolones were detected in areas not under anthropic influence, particularly at the sources of the rivers, where the number of *E*. *coli* was low. In contrast, resistance to ampicillin did not vary significantly throughout the course of the rivers. As previously described, we confirmed that human activities (housing, farming) increase the number of total *Enterobacteriaceae* and of *Enterobacteriaceae* resistant to 3GC and ciprofloxacin [32,33].

Although the WWTPs tested were in the expected range for their ability to reduce the level of total bacteria and ARC present in the raw effluent, the numbers of total bacteria and ARC found in the surface water at the discharge and downstream in river at Site #1 were nevertheless very important. The mean number of ARE strains was even higher in surface waters near the discharge than in the WWTP influents and effluents which suggests that WWTP is not the only source of ARE at Site #1. Similarly to our results, high levels of ARC were previously reported downstream of WWTP discharges in rivers [34–36], even far away from the discharge [36]. The discharge of Site #1 was located near the mouth of the river; although the number of total bacteria and ARC were high until the rivers reached the sea, they decreased rapidly in samples carried into the sea. Similarly, the numbers of *E*. *coli* and ARC in surface waters collected at the WWTP 2 discharge were similar to those observed in surface waters collected at Site #3 not impacted by WWTPs effluents. This is likely because of the higher dilution and reduced survival of cultivable bacteria in the sea. However, the risk for human infection by consumption of shellfishes or swimming may persist [18,37].

ESBLE were mostly isolated from WWTP influents and effluents, particularly from WWTP 2 that receives a direct input of resistant bacteria from the university medical hospital. This was also the case for *qnr* genes, which were only found in CTX-M15-producing *K*. *pneumoniae* and *E*. *cloacae* isolated from WWTP 2. Numerous studies have reported that WWTPs (especially those treating hospital waste water) are important suppliers of ESBL and *qnr*-producing *Enterobacteriaceae* [19,20,38,39]. The presence of ESBLE in the WWTP effluent demonstrates the inefficiency of wastewater treatment in eliminating resistant bacteria. ESBLE persistent in WWTP effluents can spread into the local environment, as demonstrated by their isolation in samples taken from discharging points and from surface waters downstream of the WWTP discharge in our study. Recent studies have also reported the presence of ESBLE in rivers and the sediments of rivers receiving WWTP discharge [18,40,41].

Most ESBLs were CTX-M variants, predominantly CTX-M-1 and CTX-M-15. The predominance of CTX-M group 1 is in agreement with the results of a recent study performed on urinary tract infections (UTI) in Guadeloupe [42]. Nevertheless, in community-acquired UTI, *K*. *pneumoniae* was the most frequent species among ESBLE strains, whereas in water samples, ESBLE were mostly *E*. *coli*. This difference is likely related to the fact that strains isolated in our study were of faecal origin, and *E*. *coli* are the predominant *Enterobacteriaceae* in faeces. Previous studies also reported *E*. *coli* as the most frequent ESBLE species detected in WWTP effluents and surface waters affected by WWTP effluents [41,43–45]. Most of these *E*. *coli* strains isolated from surface waters were probable commensal bacteria as suggested by the distribution of phylogenetic groups in our study. The predominance of groups A (50%) and D (24%) was similar to that of *E*. *coli* of faecal origin, whereas in UTIs, the phylogenetic groups B2 and D were largely predominant [46]. Most *E*. *coli* in the B2 phylogenetic group belonged to the clone O25b-ST131. In our study, this clone was not found to be related to the diffusion of ESBL, although this clone has extensively contributed to the worldwide dissemination of CTX-M-15 and has been detected in WWTP effluents, as well as in rivers subjected to heavy anthropogenic pressure [47]. The reasons for the wide dissemination and expansion of this clone remain unclear, but may include increased transmissibility, greater ability to colonise and/or persist in the human intestinal tract, enhanced virulence, and more extensive antimicrobial resistance than other *E*. *coli* strains [11]. This may explain its prevalence in the WWTPs.

## Conclusions

Antimicrobial resistant bacteria were found in different type of surface waters in Guadeloupe. Although all human activities can supply antibiotic-resistant bacteria, this study provides evidence that WWTPs were unable to eliminate antibiotic resistant bacteria and were the most important suppliers of ESBLE, susceptible to spread in surface waters in Guadeloupe. Most ESBLs belonged to CTX-M group 1 as expected. *E*. *coli* were mainly from commensal groups A and D, suggesting a faecal origin for these strains. Although the virulent clone O25b-ST131 was present, it was not related to the diffusion of ESBLs in our study. Presence of ESBLE in surface water downstream WWTPs is of great concern for public health, highlighting the need to improve hygienic measures to reduce the load of discharged bacteria.
